# A Gene Regulatory Network Model for Floral Transition of the Shoot Apex in Maize and Its Dynamic Modeling

**DOI:** 10.1371/journal.pone.0043450

**Published:** 2012-08-17

**Authors:** Zhanshan Dong, Olga Danilevskaya, Tabare Abadie, Carlos Messina, Nathan Coles, Mark Cooper

**Affiliations:** DuPont Pioneer, Johnston, Iowa, United States of America; USDA-ARS, United States of America

## Abstract

The transition from the vegetative to reproductive development is a critical event in the plant life cycle. The accurate prediction of flowering time in elite germplasm is important for decisions in maize breeding programs and best agronomic practices. The understanding of the genetic control of flowering time in maize has significantly advanced in the past decade. Through comparative genomics, mutant analysis, genetic analysis and QTL cloning, and transgenic approaches, more than 30 flowering time candidate genes in maize have been revealed and the relationships among these genes have been partially uncovered. Based on the knowledge of the flowering time candidate genes, a conceptual gene regulatory network model for the genetic control of flowering time in maize is proposed. To demonstrate the potential of the proposed gene regulatory network model, a first attempt was made to develop a dynamic gene network model to predict flowering time of maize genotypes varying for specific genes. The dynamic gene network model is composed of four genes and was built on the basis of gene expression dynamics of the two late flowering *id1* and *dlf1* mutants, the early flowering landrace Gaspe Flint and the temperate inbred B73. The model was evaluated against the phenotypic data of the *id1 dlf1* double mutant and the *ZMM4* overexpressed transgenic lines. The model provides a working example that leverages knowledge from model organisms for the utilization of maize genomic information to predict a whole plant trait phenotype, flowering time, of maize genotypes.

## Introduction

Flowering time is a major adaptive trait in plants and an important selection criterion in plant breeding [1]. Because this trait is the single most important control of the plant demand for resources and the determinant of the plant's ability to capture resources for growth, understanding the underlying genetic controls is of importance to determine efficient molecular selection strategies. Shoot apical meristem transition from the vegetative to the reproductive stage is controlled by a genetic program that is regulated by environmental and endogenous factors [2], [3]. Major components of the genetic control system for the flowering time in *Arabidopsis thaliana* have been defined in the past decades. Genetic Regulatory Network (GRN) models for flowering time control in *Arabidopsis* have been developed and often presented in graphical form [4]–[47]. Studies in other species [8]–[16], including maize (*Zea mays* L.) [17]–[19], suggest that the basic genetic components of the GRN controlling the floral transition from the vegetative to the reproductive stage are largely conserved.

The understanding of the genetic control of flowering time in maize has advanced significantly in recent years, especially after the completion of the maize genome sequence [17],[19]–[21]. Many of the flowering time pathways and genetic elements in these pathways discovered in *Arabidopsis* and rice (*Oriza sativa* L.) are conserved in maize. Through comparative genomics, mutant analysis, genetic analysis and Quantitative Trait Locus (QTL) mapping and cloning, and transgenic approaches, more than 30 flowering time candidate genes have been identified in maize. Despite these advances in molecular mechanisms, a synthesis in the form of a GRN in maize is lacking.

The limited understanding of the genetic controls of flowering time in maize in the past decades led to the development of quantitative empirical models that use environmental and genotypic rather than genomic information to predict the floral transition and the timing of pollen shedding and silking in maize. Heat units or growing degree days to shedding and to silking are examples of empirical models widely used to synchronize shedding and silking events in seed production [22]–[24]. When these models were embedded within comprehensive physiological frameworks such as CERES [25] and APSIM [26] they were applied to understand the physiological basis of maize adaptation in different environment types, construct trait performance landscapes, and predict responses to trait selection in breeding programs [27].

Empirical models such as the heat unit model have limitations to predict flowering time for novel genotypes. The advancement in the understanding of the genetic control of flowering time in maize, the availability of GRNs for model organisms, and the conservation of the main components of these GRNs across species suggest the opportunity to build upon models developed for *Arabidopsis* and rice [28]–[30] to predict flowering time in maize for existing and novel genotypes in diverse environments.

The purpose of this paper is to develop a simple model that will serve as a foundation for Dynamic Gene Network (DGN) modeling of the vegetative to reproductive transition in maize. The overall objectives of this study are: (1) to develop a conceptual model in the form of a GRN of flowering time control in maize, (2) to translate the conceptual GRN model into a quantitative DGN model, and (3) to demonstrate and evaluate the prediction of flowering time of maize genotypes varying for specific genes. First, a GRN is proposed based on a synthesis of the literature for flowering time candidate genes and their interactions. Second, a quantitative DGN model is described. Third, the DGN model is evaluated against field experimental data for flowering time of novel genotypes created from allelic variation for specific genes and from expression of transgenes.

## Materials and Methods

### Plant materials and trait phenotypes

A segregating population (*id1*/+ *dlf1*/+) for *dlf1* and *id1* mutant alleles was constructed by crossing the heterozygous *dlf1*/+ plants to the heterozygous *id1-m1*/+ plants in the B73 genetic background. Heterozygous plants were identified by the PCR genotyping method [31] and self pollinated for generating the homozygous *id1* and *dlf1* single mutants and the *id1 dlf1* double mutant. Leaf tissues of the offspring plants were taken around the V8–V10 stage for genotyping. The genotypes of individual plants were confirmed by PCR genotyping. Construction of the *ZMM4* transgenic lines in the B73, the *dlf1* and *id1* single mutant genetic backgrounds was described in detail by Danilevskaya *et al*. [32].

In order to obtain total leaf number (TLN) observations, plants of different genotypes grown in field conditions at the Pioneer Johnston research farm were tagged. The fifth leaf and the tenth leaf, and sometimes the fifteenth leaf, of the tagged plants were identified by cutting the respective leaf tips during the first half of the growing season. TLN observations of all tagged plants were obtained at or after flowering. TLN data for plants with the same genetic composition were combined and the mean and standard error statistics were estimated.

### Tissue sampling and mRNA expression measurement

Plants of the *id1* and *dlf1* mutants, the Gaspe Flint landrace, and the B73 inbred for tissue sampling were grown in a greenhouse at 25°C under 16-h day length. The V-stages were determined based on the topmost liguled leaf. Tissue samples of shoot apices were taken from the emergence stage for the Gaspe Flint landrace or the V1–V3 stage for the mutants and the B73 inbred until about one week after flowering. The intervals between two sampling times and the total number of sampling times were determined by genotypes and developmental stages. Total RNA was isolated with TRIzol Reagent in combination with Phase Lock gel. The *ZMM4* mRNA expression levels were measured by the GenomeLab GeXP analysis system at Althea Technologies. The raw RNA expression data were normalized against *α-tubulin* as the internal control within the same reaction. More details were described in Danilevskaya *et al.* [32].

## Construction of the Conceptual Gene Regulatory Network

A large number of QTL mapping studies for maize flowering time have demonstrated the complexity of the genetic architecture of this trait [20],[33]–[35]. In contrast to *Arabidopsis*, for which more than 100 flowering time genes were characterized [4],[6], only a few QTLs and mutants have been cloned in maize, and a number of homologs from other species have been identified through comparative genomics. This limited knowledge constrains our ability to fully define the topology of a GRN for flowering time in maize. The consensus GRN for *Arabidopsis* [4],[6] could be used as a scaffold to organize the limited knowledge in maize into a first logical synthesis. Using the framework provided by the *Arabidopsis* GRN, maize flowering time candidate genes were organized by pathways (Table 1) and discussed below.

**Table 1:** Candidate genes for flowering time control in maize.

Pathway	ZmGene	AtGene	Tissues Expressed	Chr	GenBank Accession	Citations
Light transduction	*PhyA1*	*PHYA*	Leaves	1.10	AY234826	[37],[114]
Light transduction	*PhyA2*	*PHYA*	broad expression	5.01	AY260865	[37],[114]
Light transduction	*PhyB1*	*PHYB*	Leaves	1.03	AY234827	[37],[38]
Light transduction	*PhyB2*	*PHYB*		9.05	AY234828	[37],[38]
Light transduction	*PhyC1*	*PHYC*	meristem, leaves	1.10	AY234829	[37]
Light transduction	*PhyC2*	*PHYC*	broad expression	5.01	AY234830	[37]
Light transduction	*ZmHy2*	*HY2*	meristem, broad expression	8.06	NM_001111786	[39]
Clock	*GIGZ1A*	*GI*	broad expression	8.03	BK006299	[49]
Clock	*GIGZ1B*	*GI*	broad expression	3.03	BK006298	[49]
Clock	*LUX*	*LUX*		8.06		[44]
Clock	*ZmCCA1*	*CCA1*	Leaves	10.04	HM452304	[48]
Clock	*ZmFKF1a*	*KFK1*	broad expression	4	NM_001152685	[43]
Clock	*ZmFKF1b*	*KFK1*	broad expression	2	EU954587	[43]
Clock	*ZmHD6*	*CK2α*	broad expression	2.10	EF114229	[115]
Clock	*ZmLHY1*	*LHY*		10.03	NM_001138057	[33],[34],[43]
Clock	*ZmLHY2*	*LHY*		4.05	NM_001154010	[33],[34],[43]
Clock	*ZmPRR37*	*PRR3*		7	EU952111	[34],[43]
Clock	*ZmPRR59*	*PPR9*		10	HQ003893	[33],[34],[43]
Clock	*ZmPRR73*	*PRR7*		9	EU952116	[43],[44]
Clock	*ZmTOC1*	*TOC1*		10.04	HM452303	[48]
Photoperiod	*COL1*	*COL1*		9.03	AC189064	[44]
Photoperiod	*CONZ1*	*CO*	broad expression	9.03	NM_001127250	[49]
Photoperiod	*ZmCCT*			10		[50]
Photoperiod	*ZCN8*	*FT*	Leaves	8.03	NM_001112776	[19]
Autonomous	*FCA*	*FCA*		2.06		[44]
Autonomous	*ID1*			1.08	NM_001111439	[62],[63]
Autonomous	*LDL1*	*LDL1*		10.03		[44]
Autonomous	*VGT1*			8.06		[94]
Autonomous	*ZmLD*	*LD*	broad expression	3.05	AF166527	[65]
Aging	*GL15*	*AP2*	meristem, leaves	9.03	NM_001112420	[116]
Aging	*miR156*	*miR156*				[73] [74]
Aging	*miR172*	*MiR172*				[73],[76],[78],[117]
GA	*D8*	*GAI*		1.09	NM_001137157	[82]
GA	*D9*	*GAI*		5.00	DQ903073	[80]
GA	*GA2ox1*	*GA2ox1*			NM_001158585	[84]
GA	*KN1*		Meristem	1.10	NM_001111966	[84]
Integrator	*DLF1*	*FD*	Meristem	7.06	NM_001112492	[31]
Integrator	*ZAP1*	*AP1*	Meristem	2.10	NM_001111863	[96]
Integrator	*ZAP1b*	*AP1*	Meristem	7.00	NM_001111457	[87]
Integrator	*ZCN2*	*TFL1*	Meristem	4.05	NM_001112770	[86]
Integrator	*ZFL1*	*LFY*	Meristem	10.06	NM_001111731	[95]
Integrator	*ZFL2*	*LFY*	Meristem	2.02	AY179881	[95]
Integrator	*ZMM4*		Meristem	1.10	NM_001111681	[32]
Integrator	*ZMM5*	*SOC1*	broad expression	9.07	NM_001111682	[87],[118]
Integrator	*ZmRAP2.7*	*TOE1*	Roots	8.06	EF659468	[77]

### Light transduction

Light is an important environmental signal implicated in the regulation of flowering time of plants. In maize, early flowering of many temperate inbred lines is associated with reduced response to light [36]. Phytochromes are the primary red/far-red photoreceptors, with three pairs discovered in maize, *PHYA1/2*, *PHYB1/2*, and *PHYC1/2* [37]. *phyB* mutants flower earlier than lines that carry functional copies of *PHYB1* and *PHYB2*. *PHYB* genes were implicated in the perception and transduction of photoperiod, thus playing a role in the delay of floral transition and flowering time under long day conditions [38]. The gene *ZmHY2* (Table 1), homologous to the *Arabidopsis HY2* gene, encodes a phytochromobilin synthase [39]. A point mutation in this gene, *i.e.*, *elongated mesocotyl1* mutant, prevents synthesis of the phytochrome chromophore and is light insensitive and exhibits early flowering [40].

### Circadian clock

Many physiological processes in plants are regulated to match daily and seasonal external changes through the endogenous timekeeper known as the circadian clock [41],[42]. The molecular mechanism of the circadian clock is largely preserved across plant species [8] [42]–[46]. Because of a recent polyploidization event that resulted in duplications of a large number of maize genomic segments [47], there are multiple copies of homologues of the *Arabidopsis* circadian clock core genes in the maize genome. Studies show that 10–23% of expressed transcripts in maize exhibit diurnal oscillations [43],[44]. The maize circadian clock regulates genetic networks controlling key physiological processes, such as carbon fixation, cell wall synthesis, phytohormone biosynthesis, flowering time, and phototropism [44].

Candidate genes in the core oscillator of the maize circadian clock include *ZmCCA1*, *ZmLHY*, *ZmTOC1a*, *ZmTOC1b*, *ZmPRR73*, *ZmPRR37*, *ZmPRR59*, *GIGZ1a*, *GIGZ1b*, *ZmFKF1a* and *ZmFKF1b*, which are homologous to their counterparts in *Arabidopsis* and rice (Table 1). The diurnal expression patterns of these key components at the mRNA and protein levels are largely conserved across plant species . A detailed study of *ZmCCA1* and *ZmTOC1* confirmed that they are the key components in the maize circadian clock [48].

### Photoperiod transduction pathway

To date, a few candidate genes involved in the photoperiod transduction pathway of maize have been published. They are *CONZ1* (also known as *ZmCO1*), *ZmCCT* and *ZCN8* (Table 1) [19],[49],[50]. *CONZ1* and its upstream genes *GIGZ1a* and *GIGZ1b* exhibit diurnal expression patterns similar to their homologues in *Arabidopsis* and rice. Maize is able to perceive the differences in photoperiod through the distinct expression patterns of *CONZ1* in long and short days [49]. *ZmCCT* is homologous to the rice photoperiod response regulator *Ghd7* and plays a critical role in maize photoperiod response [50]. Teosinte *ZmCCT* alleles are consistently expressed at higher level and confer later flowering than temperate maize alleles under long day condition. *ZCN8* is homologous to *Arabidopsis FT* and rice *Hd3a* and *RFT1* and may function as the florigen in maize [19]. The mRNA transcript of the maize *ZCN8* exhibits strongly up-regulated diurnal oscillation in leaves under inductive short days in photoperiod-sensitive tropical lines and a weak diurnal pattern in day-neutral temperate lines. Lines of evidence suggest that ZCN8 protein moves through the phloem to the shoot apical meristem to induce transition from the vegetative to the reproductive development. Transgenic plants carrying an overexpressed *ZCN8* gene flower earlier than the wild type. Down-regulation of *ZCN8* via artificial microRNA induces a late flowering phenotype. *ZCN8* was placed downstream of *ID1* and upstream of *DLF1* [19].

The regulatory relationship between *CONZ1* and *ZCN8* in maize is unknown. Deciphering the similarity with that observed between *Hd1* and *Hd3a* in rice will help frame the flowering response to photoperiod observed in maize within the context of the external and internal coincidence models. The underlying molecular mechanism inside the external and internal coincidence models consists in the form of blue-light dependent *FKF1* and *GI* protein complex, which regulates the timing of *CO* expression (internal coincidence) and stabilization and activation of the CO protein by light (external coincidence) [51]–[53]. The *Arabidopsis CO*-*FT* module is conserved in long-day plants, such as barley (*Hordeum vulgare*), wheat (*Triticum aestivum*) and poplar (*Populus alba*) [54], while the module is altered or missing in short-day plants, such as rice [55],[56] and *Pharbitis nil* [57].

### Autonomous pathway

The maize genes *ID1* and *ZmLD* have been cloned and characterized at the molecular level, and may function in the autonomous pathway to positively regulate flowering time.

*ID1* (Table 1), a zinc finger transcription factor, is only expressed in immature leaves. It is believed to be unique in cereal crops because its homologous counterparts are only found in rice and other grass species [58]–[61] but not in *Arabidopsis*. Loss-of-function *id1* mutant produces more leaves and flowers much later with aberrant floral organs [62]. Because *ID1* expression is not altered by photoperiod and is developmentally regulated it is plausible that *ID1* works through the autonomous pathway to regulate flowering time [63]. The downstream targets of the *ID1* gene may play a role in facilitating the movement of the *ZCN8* protein through the phloem to the shoot apical meristem [64]. Alternatively, *ID1* may function in the floral induction through a *CO*/*FT* independent pathway [64].

*ZmLD* (Table 1), homologous to an autonomous gene *LD* in *Arabidopsis*, is expressed in the shoot apex and developing inflorescences in maize [65]. It may function in the autonomous pathway through some unknown mechanism in maize because there is no maize homolog of the *Arabidopsis FLC* gene, which integrates signals from the autonomous and vernalization pathways in *Arabidopsis* [66].

### Aging pathway

Higher plants experience a series of phase transitions during their life cycle. At early stages of development the transition from the juvenile phase to the adult phase is the most significant developmental event. During this transition period a plant becomes competent for reproductive development [67]. In maize, the transition from the juvenile phase to the adult phase has a significant impact on the total leaf number, which is tightly associated with flowering time [68]–[70]. The genetic module in *Arabidopsis* that governs this phase transition was named the aging pathway [6]. There are two key miRNA gene families in this pathway, namely *miR156* for suppression of and *miR172* for promotion of the phase change [71],[72]. The expression of the two miRNA families is negatively correlated, that is, *miR156* expression is higher in younger tissues while *miR172* expression is higher in adult tissues [73].

*miR156* (Table 1), as a juvenile gene, regulates the transition from the juvenile phase to the adult phase through repression of *SQUAMOSA PROMOTER BINDING PROTEIN LIKE* (*SPL*) gene expression [74]. Expression of specific members of the *miR156* gene family is repressed by a developmental regulation factor produced in leaf primordia [75]. In contrast, *miR172* (Table 1) promotes the transitions between developmental phases and is involved in specifying floral organ identity by downregulating *AP2*-like target genes, such as *GLOSSY15* (*GL15*) [70] and *ZmRAP2.7* [76]–[78].

### GA pathway

Gibberellin (GA) is an endogenous plant growth regulator that affects both growth and development. *DWARF8* and *DWARF9* (Table 1) encode proteins with SH2-like domain and DELLA domain [79],[80] and are homologous to the *Arabidopsis* gene *GIBBERELLIC ACID INSENSITIVE* (*GAI*) gene. Studies show that *DWARF8* is associated with the variation in flowering time in temperate inbred lines [81],[82] and is involved in maize climatic adaptation through selection for flowering time [83]. A gain-of-function *dwarf9-1* mutant exhibits a late flowering phenotype in maize while the same allele in transgenic *Arabidopsis* lines causes the opposite phenotype [80]. Another transcription factor gene *KNOTTED1* (*KN1*, Table 1) negatively modulates the accumulation of gibberellins through regulating the gene *GA2ox1*, which encodes for an enzyme that inactivates GA [84].

### Pathway integrators

A group of genes are responsible for the integration of all floral inductive or repressive signals and for the activation of floral organ identity genes, such as *LFY* and *AP1* in *Arabidopsis* [4]–[7]. Candidate genes in maize implicated in integrating floral signals from different pathways include *DLF1*, *ZMM4*, *ZmRAP2.7*, *ZFL1*, *ZCN2*, and *ZAP1* (Table 1) [31],[32],[77],[85]–[87].

*DLF1* (Table 1), homologous to *FLOWERING LOCUS D* (*FD*) in *Arabidopsis*, encodes a bZIP protein that mediates floral inductive signals at the shoot apical meristem in maize. Loss-of-function *dlf1* mutant flowers late, indicating that *DLF1* promotes the floral transition. Gene transcript expression analysis reveals that *DLF1* transcript increases and peaks at the floral transition, which indicates that *DLF1* is involved in a positive feedback loop to promote the floral transition [31]. In the shoot apical meristem, the DLF1 and ZCN8 proteins may form a complex, which is comparable to the FD and FT protein complex in *Arabidopsis*, to activate downstream floral organ identity genes, such as *ZMM4* [19],[88].

*ZMM4* (Table 1) is a maize MADS-box gene in the *FUL1* family that regulates floral transition in temperate cereals [89]. Through double mutant analysis, *ZMM4* is positioned functionally downstream of the flowering time genes *DLF1* and *ID1*. Analysis of overexpressed transgenic lines indicates that *ZMM4* promotes floral transition and inflorescence development in maize [32]. Its mRNA expression initiates in leaf primordia of the vegetative shoot apices, increases during the elongation of the shoot apical meristem, peaks around the time of the spikelet branch meristem initiation, and then declines as inflorescence development progresses [32]. The precise regulatory mechanism of the *ZMM4* gene expression is still elusive. It could involve positive and negative feedback loops, which may be comparable to the feedback loops among the *LFY*, *AP1/FUL1* and *CAL* genes in *Arabidopsis* [32],[90],[91].

*ZCN2* (Table 1), homologous to the *Arabidopsis TFL1*, is a member of the maize *PEBP* gene family [92]. It acts as a maintainer of meristem indeterminacy. Overexpression of *ZCN2* causes delayed flowering and altered inflorescence architecture [86].

*ZmRAP2.7* (Table 1), homologous to the *Arabidopsis TARGET OF EAT1* (*TOE1*), is a negative regulator of flowering time in maize [Bibr pone.0043450-Salvi2] [77,93]. The flowering time QTL *VGT1* functions as a *cis-*regulatory element of the *ZmRAP2.7* gene by down-regulating its mRNA transcript abundance [77]. *VGT1* is mapped to chromosome arm 8L in the cross of the Gaspe Flint landrace and the N28 inbred. The Gaspe Flint allele reduces the flowering time, number of leaves, and plant height in the N28 background [94].

*ZFL1* and *ZFL2* (Table 1) affects the floral transition time and development in maize in a similar manner to their homolog *LEAFY* found in *Arabidopsis* [85]. Double mutant analysis shows that *ZFL1* and *ZFL2* act as upstream regulators of the ABC floral organ identity genes [85]. Association mapping results show that *ZFL1* is strongly associated with flowering time [95].

*ZAP1a* (Table 1) is homologous to the floral homeotic gene *AP1* in *Arabidopsis* [96] and *ZAP1b* (known as *ZmMADS3*, Table 1) is orthologous to *ZAP1a* [87]. The *ZAP1* expression pattern is restricted to terminal and axillary inflorescences and it is consistent with that observed for *Arabidopsis AP1* [96]. Studies show their functions in floral organ identity and development [87],[96]. Overexpression of *ZAP1b* reduces the total leaf number and plant height [87].

### A conceptual gene regulatory network model for flowering time control in maize

**Figure 1:**
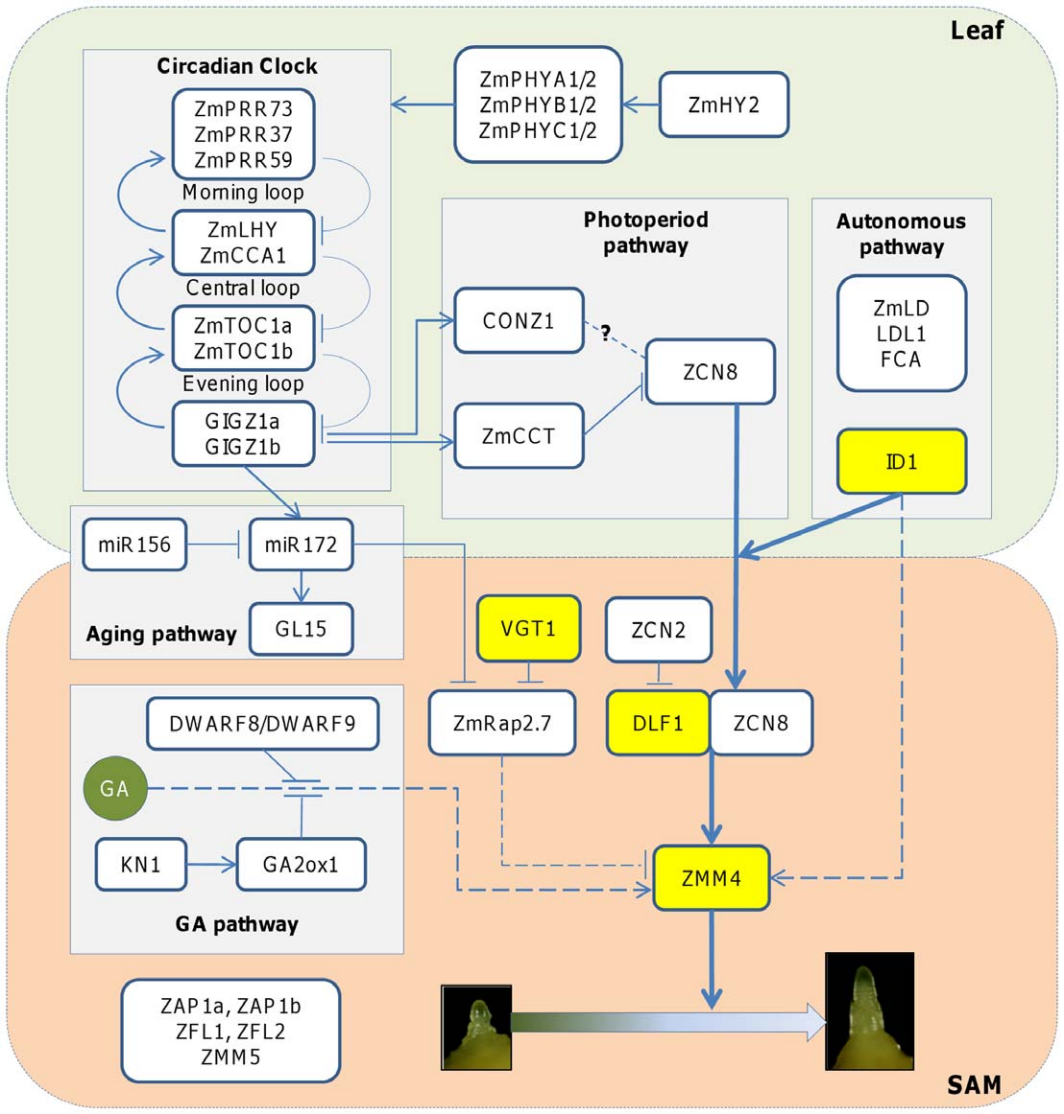
A conceptual GRN model for flowering time control in maize. The GRN model is divided into two components: leaf and shoot apical meristem (SAM). It includes several pathways: light transduction, circadian clock, photoperiod, autonomous, aging, GA pathways and pathway integration. Thick lines are confirmed by genetic analysis. Thin lines are based on comparative genomics. Arrows between genes stand for promotion or activation. T bars between genes stand for inhibition or suppression. Dashed lines stand for putative relationships derived through comparative genomics. Genes highlighted in yellow background are selected for the DGN modeling.

A conceptual GRN model for flowering time control in maize is proposed as a first synthesis of our current knowledge (Figure 1). Candidate genes are grouped into multiple pathways as described above (Table 1) based on their confirmed relationships and hypothetical relationships derived from *Arabidopsis* and rice through comparative genomics. Genes with unclear regulatory relationships are placed into the boxes without any input and output.

Upstream pathways include receptors that sense environmental cues and usually operate in leaves. Signals from the photoperiod and autonomous pathway are physically transduced from leaves to the shoot apical meristem by ZCN8 protein via movement through the phloem. Accumulation of a threshold amount of ZCN8 protein triggers the reprogramming in the shoot apical meristem which stops producing leaves and initiates the tassel development. Known key integrator genes in maize are *DLF1* and *ZMM4*.

## Dynamic Gene Network Modeling

Discrete and/or continuous DGN modeling approaches can contribute to solve the genome-to-phenome prediction problem [97]–[100]. Boolean networks, one form of a discrete DGN model, have been extensively applied to GRN models [101]–[107]. In Boolean networks, each node denotes a gene. All nodes have binary values, 0 or 1, that represent the active or inactive state of a gene. The linkages between nodes represent regulatory relationships of a gene with other genes within a given GRN. In continuous DGN modeling, a system of ordinary differential equations is employed to describe the behavior of a GRN and predict phenotypes based on the expression level of genes at the convergent point. The two approaches were combined to model gene networks around the promoter of the *endo 16* gene in the sea urchin [108]. Simple algorithms that combine logic and algebraic functions can capture major features of this promoter's behavior [109],[110]. The methodology that combines logic and algebraic functions was adapted and applied to the prediction of flowering time of maize genotypes in this study.

### A dynamic gene network model to predict floral transition time in maize

**Figure 2:**
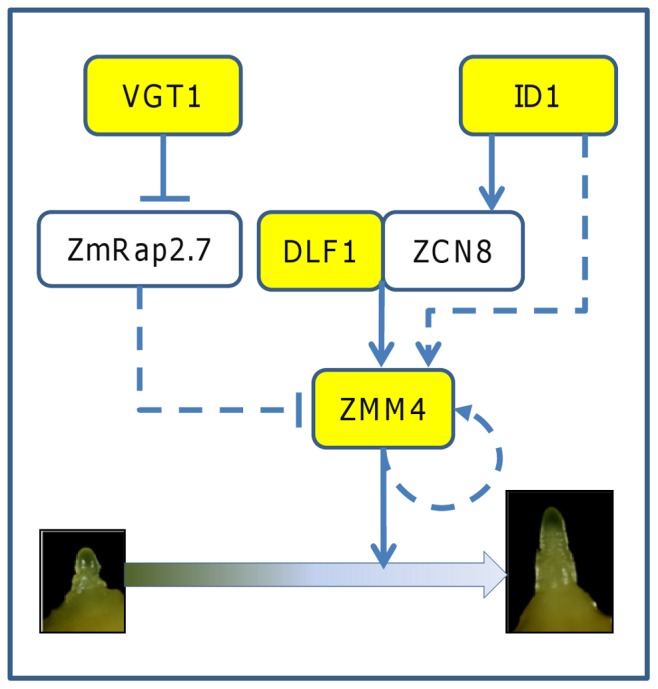
A simplified GRN model for the DGN modeling. The simplified GRN model includes the confirmed relationships through genetic analysis, putative relationships derived through comparative genomics, and the proposed *ZMM4* positive feedback loop. Arrows between genes stand for promotion or activation. T bars between genes stand for inhibition or suppression. Solid lines stand for confirmed relationships while dashed lines stand for putative relationships. Genes highlighted in yellow background are included in the DGN model.

Based on the regulatory relationships shown in the simplified GRN for maize (Figure 2), four key components of the network (*ID1*, *DLF1*, *VGT1* and *ZMM4*) were selected to develop a DGN model to predict the floral transition time in maize. The proposed DGN model includes an ordinary differential equation and can simulate the *ZMM4* mRNA expression pattern, which in turn is associated with the floral transition time of maize genotypes varying for specific genes.

The terms used to construct the differential equation model are justified here. The gene *ID1* regulates *ZMM4* expression through two paths: 1) the *DLF1*-dependent path via regulation of the ZCN8 protein movement through the phloem and 2) the direct autonomous path. Because the *ZCN8* and *DLF1* proteins combine to form a protein complex to regulate the *ZMM4* expression, the interaction between *ZCN8* and *ID1* can be substituted by a term that represents the interaction between *ID1* and *DLF1*. The model includes two regulatory terms to account for the effect of *ID1* alone on flowering time and the combined effect that results from the interaction between *ID1* and *DLF1*. The regulatory relationship between *VGT1* and *ZMM4* is through *ZmRap2.7*. The double suppression relationship can be substituted by a positive term only involving *VGT1*. As discussed earlier, there is plausible positive feedback mechanism that governs the regulation of the *ZMM4* gene expression before the floral transition and generates the exponential *ZMM4* mRNA expression pattern. A feedback term involving *ZMM4* mRNA expression is included in the model as a parsimonious approach to describe the observed growth pattern of the *ZMM4* mRNA transcript. Because all the regulatory relationships shown in the GRN (Figure 2) independently converge at the *ZMM4* node, all the terms can be added for mathematical convenience.

The presence of a gene in regulatory relationships can be expressed as a continuous quantity or as discrete binary values based on the nature of the gene and related relationships. In this study, discrete binary values, 0 and 1, for *ID1*, *DLF1* and *VGT1* were used (see detail in Table 2).

**Table 2:** Gene allele information of genotypes used in this study.

Genotype	*VGT1*	*ID1*	*DLF1*	*ZMM4*	Transgenic *ZMM4*
B73	0	1	1	1	0
*id1* mutant	0	0	1	1	0
*dlf1* mutant	0	1	0	1	0
Gaspe Flint	1	1	1	1	0
*Id1 dlf1*	0	0	0	1	0
*ZMM4* B73	0	1	1	1	1
*ZMM4 id1* mutant	0	1	0	1	1
*ZMM4 dlf1* mutant	0	0	1	1	1

**Figure 3:**
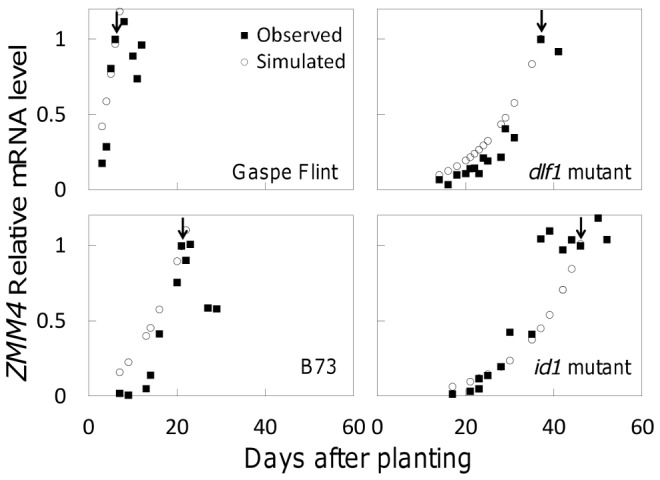
*ZMM4* mRNA expression levels in different genotypes. Scaled *ZMM4* mRNA expression levels in shoot apices before and around the floral transition in four genotypes (the Gaspe Flint landrace, the *dlf1* mutant, the B73 inbred, and the *id1* mutant) were plotted against days after planting. Filled squares are the observed mRNA expression levels whereas open circles are the predicted mRNA expression levels. The time to the floral transition of each genotype is indicated by arrows.

The peak of the *ZMM4* mRNA expression in shoot apices synchronizes well with the floral transition and is consistent across the genotypes (Figure 3). Therefore, the *ZMM4* gene will be used as a marker to indicate the floral transition and further to associate its expression level with the whole plant trait phenotype, days to floral transition or tassel initiation (DTI). In the final form of the DGN model, the *ZMM4* mRNA expression level (mZMM4) was directly associated with the floral transition status (FTS) of the genotypes under investigation as follows.
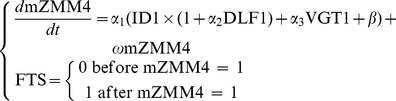
(1)where, mZMM4 stands for the mRNA expression level of the *ZMM4* gene. ID1, DLF1, and VGT1 stand for allele status of the *ID1*, *DLF1*, and *VGT1* genetic elements (Table 2). FTS stands for the floral transition status; 0 indicates the floral transition has not occurred while 1 indicates the floral transition has been reached. The floral transition time or DTI is the number of days from planting to when FTS equals 1. The coefficients, *α_1_*, *α_2_*, *α_3_*, *β*, and *ω*, are parameters in the model, which represents the strength or weight of the gene effects or the *ZMM4* feedback effect.

### Parameterization of the dynamic gene network model

Four genotypes with contrasting flowering time phenotypes were chosen to parameterize the model. They are the extreme early flowering landrace Gaspe Flint, which carries the early flowering allele of the QTL *VGT1*, the temperate inbred line B73 and the late flowering homozygous *id1* and *dlf1* mutants.

Levels of the *ZMM4* gene expression were scaled to a range of 0.0 to 1.0 across all genotypes before and at the floral transition. The *ZMM4* expression levels and DTI were used to parameterize the DGN model in Eq. 1. The multi-target objective function used in the optimization is shown in the Eq.2.
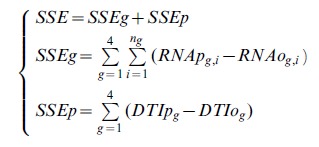
(2)where, *RNAp_g,i_* and *RNAo_g,i_* stand for predicted and observed *ZMM4* mRNA expression levels at the *i*th sampling time for the *g*th genotype; *DTIp_g_* and *DTIo_g_* stand for predicted and observed DTI for the *g*th genotype; *g* is a loop variable for genotypes and its value ranges from 1 to 4; *i* is a loop variable for the sample time (*n_g_*) of each genotype; *SSEg*, *SSEp*, and *SSE* stand for sum of squared errors for gene expression data, phenotypic data, and the sum of both, respectively. The Euler integration method was employed to numerically integrate the differential equation model in Eq. 1. The time step used in the numerical integration was 0.01 d. The mZMM4 initial value was set to zero to reflect the negligible size of the plant at t = 0.0 d. The Nelder-Mead downhill simplex method [111],[112] was used to estimate the model parameters.

Table 3 lists the estimated parameter values. The coefficient of *ID1* is assumed to be 1, the parameter *α_2_* stands for the impact of *ID1* and *DLF1* combination while the parameter α_3_ stands for the impact of *VGT1* alone. The parameter *β* is considered as the basal synthesis rate of the *ZMM4* gene. By comparing the values of the coefficients, it is evident there is a large impact of *VGT1* relative to the impact of *DLF1* and *ID1* combination and the impact of *ID1* alone. The parameter *ω* has a positive value that indicates a positive feedback loop reinforced by other integrators at a switching point before the floral transition. The parameter *α_1_* is a scaling factor that influences the size of the gene effects, including the basal synthesis of the *ZMM4* gene, relative to the effect of the positive feedback loop. Thus, the smaller value of the parameter *α1* relative to that of the parameter *ω* indicates the strong effect of the positive feedback loop.

**Table 3:** Estimated model parameter values.

Parameter name	Estimated values
α1	0.002000
α2	6.489431
α3	53.204799
β	0.821720
ω	0.086782

The predicted and observed *ZMM4* mRNA expression patterns match with each other well (Figure 3). Furthermore, the predicted DTIs match the observed, indicated by arrows in Figure 3.

### Evaluation of the dynamic gene network model

**Figure 4:**
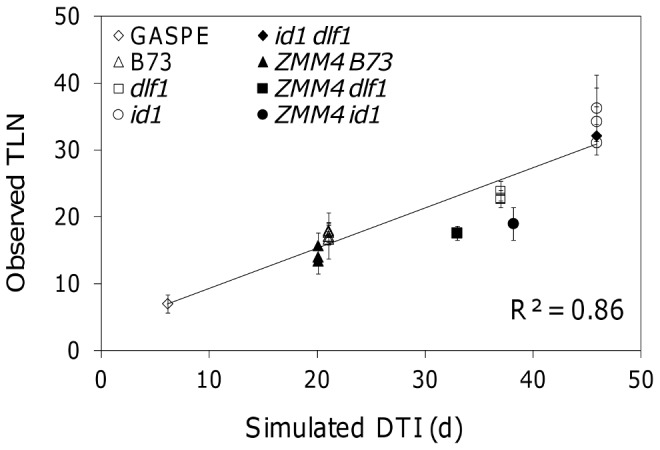
The observed total leaf number (TLN) vs the predicted days to tassel initiation (DTI). Different shapes represent different genotypes: open diamonds for the Gaspe Flint landrace, filled diamonds for the *id1 dlf1* double mutant, open triangles for the B73 inbred line, filled triangles for the *PRO_ubi_*:*ZMM4* B73 transgenic line, open squares for the *dlf1* mutant, filled squares for the *PRO_ubi_*:*ZMM4 dlf1* transgenic line, open circles for the *id1* mutant, and filled circles for the *PRO_ubi_*:*ZMM4 id1* transgenic line. The vertical bar of each point represents the standard error of the observations for a given genotype. The straight line represents the best linear fit between the observed TLN and the predicted DTI.

Novel genotypes, defined here as genotypes not used to parameterize the model, were utilized to evaluate the capacity of the DGN model to predict floral transition. Although the DGN model was developed to predict the time of transition of the shoot apical meristem from the vegetative to the reproductive stage, the model was evaluated based on observations on TLN. The rationale for this is that TLN is easier to measure and a more stable measurement across environments than DTI [113]. The process that links these two phenotypes is the rate of the leaf differentiation within the shoot apical meristem prior to the transition to the reproductive stage. Thus, the number of leaves present in the mature plants provides an accurate quantitative measurement of the time to floral transition. Phenotypic data for TLN were collected for the *id1 dlf1* double mutant created in the B73 genetic background, single mutants alone, the wild type, and the *PRO_UBI_:ZMM4* overexpressed lines in the B73, *dlf1* and *id1* mutant genetic backgrounds (Figure 4). All data were collected in the same field conditions at a single location [32].

Predictions for all genotypes except for the *PRO_UBI_:ZMM4* transgenic lines were made by using the DGN model (Eq. 1) as parameterized above. Because the *PRO_UBI_:ZMM4* transgenic lines overexpressed *ZMM4* cDNA by means of the maize constitutive *ubiquitin* promoter, significantly higher levels of the *ZMM4* mRNA transcript are expected than in non-transgenic lines. To accommodate the constitutive expression of the transgenic *ZMM4* gene, a conservative assumption was made to predict DTI for the *PRO_UBI_:ZMM4* transgenic lines. The coefficient *β*, the *ZMM4* basal synthesis rate (Eq.1), was multiplied by 2 to represent the expression of two copies of the *ZMM4* gene, the native and the transgenic copies.

The correlation between the predicted DTI and the observed TLN (R^2^ = 0.86, Figure 4) is comparable to what Russell and Stuber observed in the field for a diverse set of maize genotypes (R^2^ = 0.87) [113]. The DGN model derived from a data set of single mutants can predict the trait phenotype of novel genotypes, such as the double mutant and the overexpressed transgenic lines. This result is encouraging but limited to the prediction of effects of the selected genes.

Assuming that the *ZMM4* gene regulates the floral transition through the timing of the transition but not the rate of the leaf initiation, the predicted DTI of the *PRO_UBI_:ZMM4* transgenic lines should randomly scatter around the fitted line in Figure 4. However, all but one predicted DTI of the *PRO_UBI_:ZMM4* transgenic lines are under the fitted line. This implies the model overestimated DTI for the *PRO_UBI_:ZMM4* transgenic lines. We attribute this less accurate predicted result to an inadequate assumption about the expression level of the transgenic *ZMM4* gene under the maize *ubiquitin* promoter. Multiplying the coefficient *β* by 2 most likely underestimated the expression level of the transgenic *ZMM4* gene in the transgenic plants thus increasing the predicted DTI (Figure 4). Additional terms to accommodate effects of different promoters on gene expression could be formalized within the DGN models.

## Conclusions

This paper proposes a synthesis of our current knowledge of genetic determinants of flowering time in maize in the form of a GRN. This model can serve as a foundation to build upon as new genetic knowledge becomes available and to guide future studies. The process of model building demonstrated a realized opportunity that leveraged learning and networks created for *Arabidopsis* to organize knowledge and thoughts in a crop species such as maize. Despite different biological processes among species and processes being missing altogether in maize, the network topology identified in *Arabidopsis* provided fundamental insights to organize the knowledge created for maize. The conceptual GRN model provides the basic knowledge to conduct a rudimentary quantitative modeling exercise. The resulting DGN model is a step forward relative to current empirical models utilized to predict flowering time in maize. The performance of the simple model is encouraging and suggests there is an opportunity to develop quantitative models that transparently map genes and their effects to whole plant phenotypes. Numerous paths could be foreseen to advance this quantitative model with disparate objectives: from simply advancing our understanding of flowering time in maize, to the study of the emergent properties of GRN models, to facilitation of gene discovery, maize breeding and transgenic product development.
